# Demethylcalabaxanthone from *Garcinia mangostana* Exerts Antioxidant Effects through the Activation of the Nrf2 Pathway as Assessed via Molecular Docking and Biological Evaluation

**DOI:** 10.3390/antiox12111980

**Published:** 2023-11-07

**Authors:** Simona De Vita, Milena Masullo, Sabrina Grambone, Paloma Bermejo Bescós, Sonia Piacente, Giuseppe Bifulco

**Affiliations:** 1 Department of Pharmacy, University of Salerno, Via Giovanni Paolo II 134, 84084 Fisciano, Italy; sdevita@unisa.it (S.D.V.); mmasullo@unisa.it (M.M.); s.grambone@studenti.unisa.it (S.G.); 2 Departamento de Farmacología, Farmacognosia y Botánica, Universidad Complutense de Madrid, 28040 Madrid, Spain; bescos@ucm.es

**Keywords:** xanthones, *Garcinia mangostana*, antioxidant activity, molecular docking

## Abstract

Nuclear factor erythroid 2-related factor 2 (Nrf2) pathway activation promotes the expression of antioxidant enzymes in response to rising oxidative stress, resulting in reactive oxygen species (ROS) detoxification and playing a central role in the maintenance of intracellular redox homeostasis and regulation of inflammation. Moreover, the biological effects of Nrf2 pathway activation contribute to reducing apoptosis and enhancing cell survival. The activity of Nrf2 is negatively regulated by Kelch-like ECH-associated protein 1 (Keap1). Prompted by the recent results reporting the impact of xanthone metabolites on oxidative stress, cancer, and inflammation, the antioxidant properties of xanthones isolated from *Garcinia mangostana* (γ-mangostin, α-mangostin, 8-deoxygartanin, demethylcalabaxanthone, garcinone D) were assessed. In particular, the capability of these natural products to disrupt the interaction between Kelch-like ECH-associated protein 1 (Keap1) and nuclear factor erythroid 2-related factor 2 (Nrf2), triggering the activation of the Nrf2-mediated pathway, was evaluated using molecular docking experiments and in vitro tests. The modulation of some key Nrf2-related mediators like glutathione (GSH) and lactate dehydrogenase (LDH) to highlight a possible direct antioxidant effect was investigated. Among the tested compounds, demethylcalabaxanthone showed an indirect antioxidant effect, as corroborated by a Western blot assay, displaying a significant increase in the translocated protein upon its administration.

## 1. Introduction

Oxidative stress involves a wide variety of physiological and physiopathological processes, both endogenous and exogenous, that affect cellular homeostasis [1] and occur when reactive oxygen species (ROS) production exceeds the antioxidant defense systems [2]. ROS are by-products of the oxidative phosphorylation process and are mainly represented by superoxide anion (O_2_^−^), hydroxyl radical (OH), and hydrogen peroxide (H_2_O_2_) [3], which generate oxidative alteration of biological macromolecules such as proteins, lipids, and nucleic acids [4,5]. To face these harmful substances, intrinsic antioxidant response mechanisms have been developed [6]. Some of the key players in the regulation of the antioxidant response are the nuclear factor erythroid 2-related factor 2 (Nrf2) and its natural inhibitor, the Kelch-like ECH-associated protein 1 (Keap1) [2]. Nrf2 belongs to a group of specialized transcription factors, the cap-‘n’-collar (CNC) subfamily, and, more specifically, it is a basic-region leucine zipper (bZIP) [7]. Its role goes beyond ROS detoxification because it is involved in cellular homeostasis, cell growth regulation, apoptosis, and inflammatory response, and the ubiquitin-mediated degradation pathway [8]. Keap1, on the other hand, is a dimeric cytoplasmic protein that interacts with actin and keeps Nrf2 sequestered in the cytoplasm, blocking its activity. Therefore, in response to oxidative stress, Nrf2 dissociates from Keap1, moves into the nucleus, where it binds to small Maf (musculoaponeurotic fibrosarcoma) proteins, and forms the heterodimer that, eventually, activates the expression of genes containing antioxidant responsive elements (AREs) in their promoter [9]. Disrupting Keap1/Nrf2 interaction is, therefore, a promising strategy to stimulate the antioxidant response [6,10], and several natural compounds have been studied as protein–protein interaction (PPI) inhibitors [11,12,13].

Xanthones or 9*H*-xanthen-9-ones are specialized metabolites found in some higher plants. They are tricyclic polyphenols consisting of two benzene rings attached by a carbonyl group and oxygen, restricting free rotation around the carbon–carbon bonds. The name “xanthone” is a combination of “xanth”, which means yellow, and “one”, which refers to the keto group in the structure.

Some of the most famous natural xanthones are mangostins, prenylated and oxygenated xanthones, which are isolated as specialized metabolites from the mangosteen fruit (*Garcinia mangostana* L.) and have been reported to have multiple biological effects (with antioxidant, anti-inflammatory, anti-cancer, neuroprotective, hypoglycemic, anti-obesity, cytotoxic, anti-proliferative, and antibacterial properties) [14,15].

*Garcinia mangostana*, belonging to the family of Clusiaceae, is a tropical evergreen plant native to the Sunda and Maluku Islands. This plant produces a red-purple fruit well known for its nutraceutical purposes. The shell, bark, and roots of the tree have been used for hundreds of years for their biological properties, including for skin disease, bacterial infection, wound healing, and inflammation [14].

Based on the recent findings reporting the effect of xanthones on oxidative stress, cancer, inflammation, infections, and other pathologies [16,17,18], the antioxidant effects of xanthones from *G. mangostana* were assessed using molecular docking experiments and in vitro tests. The isolated compounds were tested for their ability to disrupt the interaction between Keap1 and Nrf2, triggering the activation of the Nrf2-mediated pathway. 

## 2. Materials and Methods

### 2.1. General Experimental Procedures

The nuclear magnetic resonance (NMR) experiments were carried out in methanol (MeOH)-*d*_4_ (99.95%, Sigma-Aldrich, Milan, Italy) on a Bruker Ascend-600 NMR and Bruker Ascend-400 spectrometer (Bruker BioSpin GmBH, Rheinstetten, Germany) at 300 K. Data processing was carried out with the software Topspin 3.2. Standard pulse sequences and phase cycles were used for DQF-COSY, HSQC, and HMBC spectra. RP-HPLC-UV separations were performed on an Agilent 1260 Infinity system (Agilent Technologies, Palo Alto, CA, USA), equipped with a binary pump (G-1312C) and a UV detector (G-1314B), with a Phenomenex C18 Synergi-Hydro-RP column (250 mm × 10 mm, 10 μm). HRESIMS data were acquired on an LTQ Orbitrap XL mass spectrometer (Thermo Fisher Scientific, San Jose, CA, USA) operating in negative ion mode.

### 2.2. Plant Material

The fruits of *G. mangostana* were purchased from the online market in April 2019. A copy of a voucher (No. 150) was deposited at the Department of Pharmacy of the University of Salerno.

### 2.3. Extraction and Isolation

The arils of *G. mangostana* (350 g) were stored in the freezer at a temperature of −5 °C. After few days, the fruits were subjected to lyophilization to obtain 60.5 g; the dried arils were extracted at room temperature via maceration with petroleum ether (300 mL for 3 days, three times a day) and CHCl_3_ (300 mL for 3 days, three times a day). An amount of 1.20 g of CHCl_3_ raw extract was obtained via filtration and evaporation of the solvent. The shells of *G. mangostana* (500 g) were dried and extracted at room temperature via maceration with petroleum ether (1500 mL for 3 days, 3 times) and CHCl_3_ (1500 mL, 3 times). The filtrate was concentrated under reduced pressure until the elimination of CHCl_3_ to obtain 17.50 g of raw extract. The CHCl_3_ extracts of arils and shells were purified via HPLC-UV. The elution gradient was performed using water with 0.1% formic acid as eluent A and acetonitrile with 0.1% formic acid as B, at a flow rate of 2.0 mL/min. Specifically, the HPLC gradient started at 5% B, and after 5 min, B was at 50%, after 15 min at 80%, and after 10 min at 87%. B remained at 87% for 20 min and after 10 min reached 100%, and so remained for 20 min. In brief, the chloroform extract (2.5 mg injection, 20 times) of arils afforded garcinone D (**5**) (1.4 mg, t_R_ = 24.1 min), 8-deoxygartanin (**3**) (1.3 mg, t_R_ = 32.1 min), α-mangostin (**2**) (2.9 mg, t_R_ = 35.0 min), and demethylcalabaxanthone (**4**) (1.8 mg, t_R_ = 44.7 min). From the chloroform extract (injection of 2.5 mg, 20 times) of shells, garcinone D (**5**) (2.3 mg, t_R_ = 24.1 min), γ-mangostin (**1**) (2.0 mg, t_R_ = 29.3 min), and α-mangostin (**2**) (5.2 mg, t_R_ = 35.0 min) were isolated.

The purity of the compounds (>99%) was determined via HPLC analysis.

### 2.4. Molecular Docking

The crystallographic structure of the Keap1 Kelch domain, containing a known inhibitor (PDB: 6TYM) [19], was downloaded from the Protein Data Bank and prepared with the Protein Preparation Wizard (Epik v. 5.5, Impact v. 9.0) [20]. Through this procedure, missing atoms were added, binding orders were adjusted, N- and C-termini were protected, and all molecules not related to protein function were deleted to speed up the calculation. The structures of the test compounds were designed in 3D with Maestro Build Panel software (v. 11) [21] and prepared with LigPrep (v. 5.3) [22]. In this procedure, the protonation states of the atoms at physiological pH (7.4 ± 1.0) were assigned, and all the possible tautomers/conformers were generated if not explicitly indicated in the initial structure. Finally, the molecules were minimized using the force field OPLS-2005 (optimized potentials for liquid simulations), which is helpful in reproducing the thermodynamic properties in the liquid state for a variety of small organic molecules. All molecular docking experiments were conducted using the Glide (v. 9.0) [23,24,25,26] software. The necessary grid was generated using the co-crystallized ligand as a guide. The centroid of the molecule was set as the center of the grid and the box was extended for 30 Å in the three directions of space. Molecular docking was carried out using the Virtual Screening Workflow of Glide. With this method, the molecules were initially screened with High-Throughput Virtual Screening and Standard Precision docking, preserving 50% of the total poses generated for each ligand. In the final stage, docking was carried out in the Extra-Precision mode, with 15 poses generated per ligand, keeping only the best one for each compound. The obtained poses were visually analyzed to determine their interactions with the biological counterpart.

### 2.5. In Vitro Experiments

#### 2.5.1. Cell Cultures and Treatment

The cell cultures used in this study were the human neuroblastoma cell line (SH-SY5Y) and the human embryonic kidney cell line (HEK293-*tau*), with overexpressed *tau* protein. The two cell lines were grown in Dulbecco’s Modified Eagle Medium (DMEM, Lonza, Pontevedra, Spain) as the culture medium. The culture medium was supplemented with 10% fetal bovine serum (FBS, Biowest, Nuaillé, France) and 0.5% gentamicin (10 mg/mL, Lonza, Pontevedra, Spain) for SH-SY5Y cells and Zeocin at 100 μg/mL for HEK293-*tau*. Both cell lines were grown in an incubator at 37 °C in a 5% CO_2_ atmosphere in Petri dishes (100 × 20 mm) and collected at 90% confluence.

For the antioxidant tests, SH-SY5Y and HEK293-*tau* cells were seeded into Petri dishes (60 × 15 mm) at a density of 2 × 10^6^ cells/dish and incubated for 48 h in DMEM containing 1% of FBS. They were treated with test compounds dissolved in DMSO and diluted with PBS to reach a concentration of 10 μM (non-toxic concentration) and a percentage of DMSO < 0.1%. After 30 min, 200 μM and 100 μM solutions of H_2_O_2_ were added to SH-SY5Y and HEK293-*tau* cells, respectively, to induce oxidative stress. Cells were then incubated for 24 h, collected with a sterile scraper directly from the plate, placed in a sterile tube (Eppendorf 2.0 mL), and centrifuged at 800 rpm at 4 °C for 5 min. One milliliter of the supernatant was stored in the freezer at −80 °C in another sterile tube, whereas the pellet was washed with sterile saline phosphate buffer (PBS), centrifuged again under the same conditions, and stored at −80 °C.

#### 2.5.2. Cytotoxicity Test

A solution of trypsin-EDTA was added to the Petri dish and left to act for a few minutes to detach the cells present on the bottom of the plate. Then, to stop the action of trypsin, a suitable volume (variable according to the size of the Petri dish) of complete medium was added. For the cell count, the Neubauer chamber was used. Cells were seeded into 96-well plates at 20,000 cells/well for HEK293-*tau* and 50,000 cells/well for SH-SY5Y and incubated at 37 °C for 24 h. After that, the culture medium from the plate was removed, the test compounds were added in a concentration range from 5 to 15 μM, and the plate was placed in incubation for 24 h. Eventually, 20 μL/well of a sterile MTT solution (2.0 mg/mL in 100 μL of saline phosphate buffer PBS) was added and left to incubate for 1 h. The formazan crystals formed were solubilized by adding 100 μL/well DMSO, and the fluorescence was measured at 550 nm using a Spectrostar Nanomicroplate reader (BMG Labtech, Ortenberg, Germany). The results are expressed as the percentage of cell viability, taking the mean absorbance values of untreated control cells as 100%.

#### 2.5.3. Cytoprotection against Oxidative Stress Induced by H_2_O_2_

Cells were trypsinized with a Trypsin-EDTA solution and, after a few minutes, a suitable volume of complete culture medium was added to block the action of trypsin. Afterward, the cells were counted and seeded at a density of 50,000 cells/well in 96-well plates and incubated for 24 h at 37 °C to facilitate adherence to the wells. At this point, compounds were added at a concentration of 10 μM. After 1 h of incubation, the cells were exposed to an H_2_O_2_ solution (200 μM for SH-SY5Y cells and 100 μM for HEK293-*tau* cells) for 2, 6, and 24 h, and, finally, cell viability was measured with an MTT test (MTT 2 mg/mL in saline phosphate buffer PBS). The results are expressed as a percentage of viability, taking the mean absorbance of untreated cells as 100%.

#### 2.5.4. LDH Liberation Test

This test was carried out on the supernatant resulting from the treatment and centrifugation described above (see Section 2.5.1). In a multichannel 96-well plate, 100 μL of supernatant per well, 50 μL of phosphate buffer pH 7.4 50 mM, 50 μL of a solution of 0.18 mM NADH, and 0.6 mM sodium pyruvate solubilized in phosphate buffer were added. The plate was read at the spectrofluorometer, after 1 min at 25 °C, at λ_exc_ = 340 nm, and at λ_em_ = 445 nm [27]. Data are expressed as the ratio between the absorbance value obtained from treated cells and the absorbance of the untreated cells when the LDH content is at its maximum. 

#### 2.5.5. Determination of Reduced Glutathione Level (GSH)

Cell pellets were treated with 130 μL (SH-SY5Y) and 200 μL (HEK293-*tau*) of trichloroacetic acid in redox quenching buffer (TCA-RQB) and made to undergo vortex agitation until complete dissolution of the pellet. The obtained solution was centrifuged at 12,000 rpm for 10 min at 4 °C. After centrifugation, the supernatant was separated from the pellet, and the latter was dissolved in 500 μL of NaOH 0.1 M and used to quantify the protein content using the bicinchoninic acid (BCA) method (see Section 2.5.6), whereas the supernatant was used for the estimation of glutathione. Subsequently, 15 μL (in duplicate) of treated cell supernatant and 25 μL of TCA-RQB (trichloroacetic acid in redox reaction buffer) were added to a 96-well plate untreated control. Then, 5 μL of NEM (N-ethylmaleimide) was added to the control and 5 μL of RQB was added to the wells with treated supernatant. NEM blocks glutathione in its reduced form, so it was used in control cells to set a benchmark. Afterward, 35 μL of 1 M phosphate buffer was added and the plate was agitated at room temperature for 15 min. Eventually, 150 μL of 0.1 M phosphate buffer and 20 μL of OPA (*o*-benzodialdehyde), which reacts with the reduced glutathione present, forming a fluorescent compound, were added and agitated at room temperature for 30 min. The results were read with the spectrofluorometer Fluostar Omega at λ_em_ = 460 nm and λ_exc_ = 360 nm.

#### 2.5.6. Quantitative Determination of Protein Content using the BCA Method

This is a colorimetric test used to quantify the protein amount in a sample based on comparing it with standard bovine serum albumin (BSA) solutions at known concentrations. Briefly, the protein content in the sample reduced copper from Cu^2+^ to Cu^+^ ions in a basic environment chelated by bicinchoninic acid (BCA) to form a violet-purple complex. The absorbance of the formed complex was measured at a wavelength of 550 nm, and the intensity of the color was directly proportional to the amount of protein present in the test solution.

Two solutions were prepared, with one consisting of 2.5 g of bicinchoninic acid (BCA), 5.0 g of Na_2_CO_3_ in H_2_O, 0.4 g of Na_2_C_4_H_4_O_6_·2H_2_O, 1.0 g of NaOH, and 2.375 g of NaHCO mixed in 250 mL of distilled water (defined as solution A) and the second consisting of 2.0 g CuSO_4_·5H_2_O in 50 mL of distilled water (called solution B). Solution B and A were mixed at a ratio of 1/50, respectively (solution C). The test was carried out on a 96-well multichannel plate by adding to each well different volumes of total cell extract diluted in distilled water. Finally, solution C was added. After stirring, the plate was incubated at 37 °C for 30 min and then cooled to room temperature for a few minutes. Protein concentration was read with the Spectrostar Nanomicroplate reader, as previously mentioned.

#### 2.5.7. Protein Extraction

Total cell extracts were prepared for protein analysis. A lysis buffer containing 200 μL of protease inhibitors was added to the cell pellets. The lysis solution consisted of 10 mM Tris HCl (pH 7.5), 0.l5% Chaps, 1 mM Cl_2_Mg, 1 mM EGTA, 10% glycerol, 5 mM β-mercaptoethanol, 10 mM NaF, 1 mM Na_3_VO_4_, 1 μg/mL of Leupeptin, 1 μg/mL of Pepstatin, and 1 μg/mL of Aprotinin. The lysate was homogenized for 30 s using a motor pestle (Pellet Pestle cordless motor, Kimble) and agitated for 30 min with a vortex at 4 °C to promote cellular lysis. The pellet was then centrifuged at 4 °C, 13,000 rpm, for 15 min. The supernatant obtained was used for the following protein analysis.

#### 2.5.8. Protein Dosage Using the Bradford Method

A protein solution at a known concentration (0.2 mg/mL in distilled water) was prepared and loaded in a 96-well plate at increasing concentrations (0.4 μg/mL, 0.8 μg/mL, 1.2 μg/mL, 1.6 μg/mL, 2 μg/mL, 3 μg/mL, and 4 μg/mL). After that, distilled water and Bradford dye reagent (Bradford Working Solution) were added to each well, and the plate was shaken for 5 min. The intensity of blue staining was measured spectrophotometrically at λ = 595 nm, being directly proportional to the amount of protein in the sample. The unknown protein concentration of the sample solution is the x extrapolated from the equation of the straight line, y = ax + b, obtained with the BCA method.

#### 2.5.9. Polyacrylamide Gel Electrophoresis (SDS-PAGE)

The protein samples were mixed with Simple Buffer 5X containing 60 mM Tris HCl (pH 6.8), 25% glycerol, 2% SDS, 14.4 mM β-mercaptoethanol, and 0.1% bromophenol blue, until reaching a final concentration of 1×. They were then heated to 100 °C for 5 min to encourage denaturation.

The composition of polyacrylamide gels for electrophoretic protein separation in the Western blot experiments was 0.67 mL of a mixture of acrylamide/bisacrylamide (30% and 0.8%), 2.3 mL of H_2_O, 1 mL of Tris HCl 0.5 M (pH 6.8), 4 mL of 10% SDS, 5 μL of TEMED, and 30 μL of 10% APS for the 5% stacking gel and 4 mL of a mixture of acrylamide/bisacrylamide (30% and 0.8%), 3.5 mL of H_2_O, 2.5 mL of Tris HCl 1.5 M (pH 6.8), 4 mL of 10% SDS, 10 μL of TEMED, and 50 μL of 10% APS for the 12% running gel.

After setting up the SDS-PAGE chamber, a quantity of 10 μg of protein for each sample was loaded along with the marker (Precision Plus Protein™ Dual Color Standards MW 10–250 kDa, Bio-Rad, Alcobendas, Spain) into the wells of a 12% polyacrylamide gel. Electrophoresis was performed by applying a constant amperage of 40 mA for 40 min (Mini-protean^®^ Tetra Cell, Bio-Rad), in Running Buffer 1× containing 25 mM Tris base, 192 mM glycine, and 0.1% SDS.

#### 2.5.10. Western Blotting

At the end of the electrophoretic run, the stacking gel was eliminated, and the proteins were transferred from the running gel to a nitrocellulose membrane (Bio-rad Trans-Blot^®^ Turbo™, Transfer System, Bio-Rad, Madrid, Spain). The transfer (blotting) was conducted in a transfer buffer (Trans-Blot^®^ Turbo™ 5× Transfer Buffer, Bio-Rad, Madrid, Spain), applying a difference in potential of 19 V for 7 min. The membrane was exposed to a blocking solution (5% albumin in PBS 1×) and agitated for about 2 h. Subsequently, the membrane was incubated overnight at 4 °C on an orbiting shaker, with the primary antibody of rabbit anti-Nrf2 (Nrf2 Phospho-S40 Antibody, Signalway Antibody, Greenbelt, MD, USA) diluted in PBS-Tween (0.1% Tween) 1:1000 with 5% BSA added. The anti-Nrf2 antibody (Phospho-S40) detects endogenous levels of Nrf2 only when phosphorylated in the S40 position. The membrane was washed for 30 min with PBS-Tween to remove the antibody excess. At the end of the washes, the membrane was incubated for 60 min under agitation with the appropriate secondary anti-IgG antibody of rabbit (1:20,000, Santa Cruz Biotechnology, Heidelberg, Germany) diluted in PBS-Tween and 5% of BSA. After the indicated time, other washes were carried out with PBS-Tween for 30 min and, eventually, the nitrocellulose membrane was incubated for 5 min with a detection solution ECL-enhanced chemiluminescence detection kit (Amersham™ ECL™ Select Western Blotting Detection Reagent, GE Healthcare, Madrid, Spain). The protein bands were detected using an Image Quant LAS500 (GE Healthcare, Madrid, Spain) spectrometer and analyzed using Image Quant 1D gel analysis software (v. 10.2). Once the detection was completed, the membrane was recovered to identify β-actin. To do so, the membrane was washed for 5 min with PBS-Tween to remove the ECL solution. Then, it was further washed with 5% acetic acid for 10 min and again with PBS-Tween and, finally, blocked with 10% milk in PBS for 1 h. At this point, the membrane was first exposed to the primary antibody of mouse anti-β-actin (Santa Cruz Biotechnology, Heidelberg, Germany) and then incubated with the appropriate mouse secondary anti-IgG antibody (1:40,000, Sigma-Aldrich, Madrid, Spain) as described before.

#### 2.5.11. Statistical Analysis

The described experiments were carried out in duplicate, and the evaluation of the significance of the experimental data was carried out via two-way ANOVA or one-way ANOVA, followed by testing Dunnett’s multiple comparisons or Tukey’s multiple comparisons. Data processing, statistical analysis, and graphs were obtained with the software Graphpad Prism 7 (Graphpad Software, San Diego, CA, USA).

## 3. Results and Discussion

**Figure 1:**
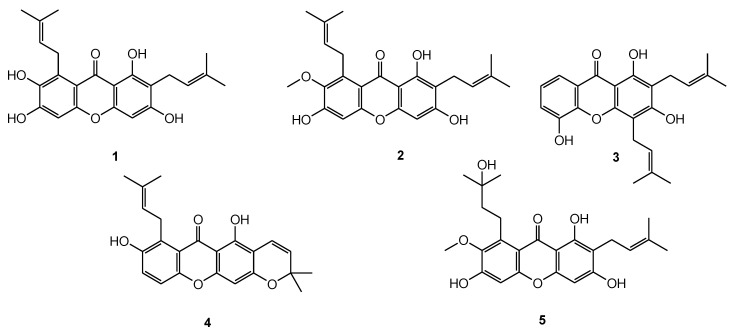
Xanthones isolated from *G. mangostana*.

The expression of antioxidant enzymes in response to rising oxidative stress is regulated by the nuclear factor Nrf2. This protein is physiologically sequestered in the cytoplasm by its specific inhibitor, Keap1, and disrupting this binding can induce the expression of the genes depending on this transcription factor. Compound **2** is reported to activate the Nrf2 pathway in retina degeneration [28], so a possible similar effect of these structurally related molecules was explored. In detail, the capability of these natural products to interact with the binding domain of Keap1 and prevent the inhibition of Nrf2 was evaluated. This study aimed at elucidating the antioxidant properties of xathones isolated from *G. mangostana* using molecular docking experiments and biological in vitro tests. In particular, the attention was focused on γ-mangostin (**1**), α-mangostin (**2**), 8-deoxygartanin (**3**), demethylcalabaxanthone (**4**), and garcinone D (**5**) (Figure 1).

### 3.1. Extraction and Isolation

Xanthones occurring in *G. mangostana* are mainly represented by prenylated derivatives. Their occurrence showed great variability according to the selected part of the fruit and was also affected by the extraction solvent [29]. Herein, the fruits of *G. mangostana* were separated into arils and shells and dried. According to our previous investigation revealing how CHCl_3_ extract, after defatting with petroleum ether, showed a high number of xanthones [29], arils, and shells were extracted sequentially with petroleum ether and CHCl_3_. The chloroform extracts were purified via HPLC-UV to obtain γ-mangostin (**1**), α-mangostin (**2**), 8-deoxygartanin (**3**), demethylcalabaxanthone (**4**), and garcinone D (**5**) (Figure 1).

### 3.2. Molecular Docking

To assess the capability of compounds **1**–**5** of interfering with the Keap1/Nrf2 complex, the 3D molecular structures of the most representative *G. mangostana* metabolites (Figure 1) were built in Maestro and processed with Ligprep [22] to assign partial charges and generate the tautomers at physiologic pH.

At the same time, the target protein, the Kelch domain of Keap1 (PDB: 6TYM) [19], was prepared for the calculations using the Protein Preparation Wizard tool of the Schrödinger Suite (Schrödinger LLC, New York, NY, USA) [20]. In this way, common structural mistakes (bond orders, atom partial charges, missing atoms, etc.) were fixed. The molecular docking studies were carried out with the Virtual Screening Workflow, which is included in the software Glide [23,24,25,26], divided into three phases with increasing precision: (i) HTVS, (ii) SP, and (iii) XP. Eventually, only one pose per ligand was kept.

The poses generated were visually analyzed to detect any interaction with Keap1 amino acids involved in the bond with Nrf2. As previously reported, the Keap1/Nrf2 binding site within the Kelch domain can be divided into five pockets [6,7]:

- P1: Arg415, Ile461, Gly462, Phe478, Arg483, and Ser508;
- P2: Ser363, Arg380, Asn382, and Asn414;
- P3: Gly509, Ser555, Ala556, Gly571, Ser602, and Gly603;
- P4: Tyr525, Gln530, and Tyr572;
- P5: Tyr334 and Phe577.

**Figure 2:**
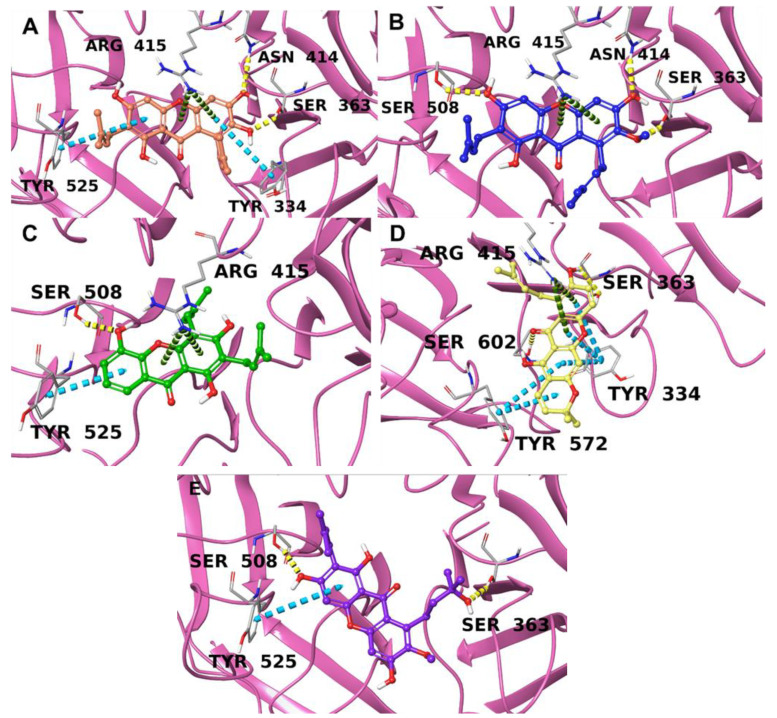
Ligand–protein interactions for **1** (**A**, in orange), **2** (**B**, in blue), **3** (**C**, in green), **4** (**D**, in yellow), and **5** (**E**, in purple) with the binding pocket of Keap1 (pink ribbons). Hydrogen bonds are indicated by yellow dotted lines, green dotted lines indicate π-cation interactions, and π-π stackings are indicated by cyan dotted lines. The interacting amino acids are labeled.

The arginine triad (Arg380, Arg415, and Arg483) is crucial for the specificity of the molecular recognition, and the hydrophobic residue group (Tyr334, Cys434, Ile461, Phe478, Tyr525, Tyr572, and Phe577) contributes to the stabilization of the complex [6,30]. The compounds showed promising results based on the number and type of interactions between the molecules and the residues belonging to the five Keap1 subpockets (Table 1 and Figure 2).

In detail, due to their chemical structure, these compounds are keen on forming hydrophobic contacts, aromatic interactions, and hydrogen bonds with the macromolecular counterpart, especially with amino acids belonging to the five subpockets, and may compete with Nrf2 for the binding site. Competitive inhibition could represent a valid strategy of promoting the Nrf2-mediated antioxidant pathway. 

### 3.3. In Vitro Experiments

The biological tests were initially carried out on the SH-SY5Y cell line, which is widely used as a model of neuronal cells, as they maintain many original biochemical and functional properties. SH-SY5Y cells are, therefore, an appropriate cell model for the neurotoxicity and neuroprotection study [31]. The second cell line, HEK293-*tau*, was introduced at a later stage due to the reliability of cell growth and stability [32]. Moreover, they are human embryonic kidney cells that overexpress the human *tau* protein. *Tau* is associated with microtubules and promotes their assembly and stabilization; under pathological conditions, called tauopathies, *tau* is modified by hyperphosphorylation and detaches from microtubules, forming aggregates. This cell model was proposed to evaluate the possible protective effect of compounds in cells different from neurons but still involved in neurodegenerative diseases.

#### 3.3.1. Cytotoxicity Test

The first step was determining the toxicity of compounds **1**–**5** on the cell lines used for the MTT test. All compounds were tested at a concentration of 10 μM, and the MTT reduction to formazan was measured via spectrophotometric reading at a wavelength of 550 nm [33]. The MTT test was carried out on both cell lines in the absence of compounds. The results show that no compound was particularly harmful towards the selected cell lines (Figure S16, Supplementary Materials).

As expected, the HEK293-*tau* cells were more sensitive to external agents than SH-SY5Y cells; however, more than 50% of cells remained alive after the treatment with compounds **1**–**5**.

#### 3.3.2. Cytoprotection against Oxidative Stress Induced by H_2_O_2_

**Figure 3:**
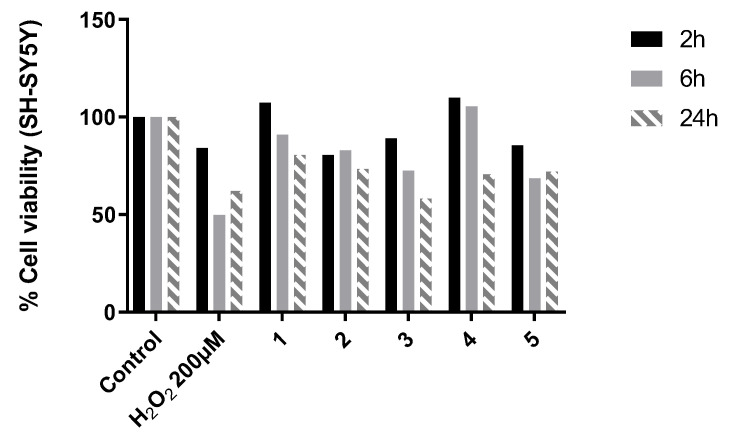
Results of the cytoprotection test of compounds **1**, **2**, **3**, **4**, and **5** expressed as the percentage of viability compared to control (100%). H_2_O_2_ (200 μM) was used as the negative control.

The intracellular overproduction of ROS contributes to cell damage and leads to oxidative stress conditions. In this scenario, it was assessed whether compounds **1**–**5**, after being proved non-toxic, were capable of exerting a protective effect on cells in an induced oxidative state. Hydrogen peroxide was chosen as the toxic agent to generate the hydroxyl radical (OH). After pretreatment with compounds **1**–**5**, SH-SY5Y cells were exposed to H_2_O_2_ (200 μM) for 30 min (Figure 3).

A 2 h incubation period was, in some cases, not sufficient for H_2_O_2_ to exert its toxicity (see H_2_O_2_ vs. control after 2 h), while at 24 h, the effect started to fade away, probably due to the restoration of the antioxidant mechanism (see H_2_O_2_ vs. control at 24 h). The values obtained after 6 h were, therefore, selected, and the compound which gave the highest protection against induced oxidative stress was **4** (105.4% vs. control). Furthermore, **1** (90.9% viability compared to cells treated with H_2_O_2_), **2** (82.9%), and **3** (72.6%) showed interesting cytoprotective properties, whereas compound **5** showed values not too different from those of H_2_O_2_ alone; therefore, its behavior did not suggest a protective action towards oxidative damage.

#### 3.3.3. Determination of Reduced Glutathione Level (GSH)

GSH (γ-glutamyl-l-cysteinylglycine) is one of the cell-essential antioxidant molecules. It works as a direct scavenger or synergistically with enzymes like glutathione peroxidases (GPx) or glutathione S-transferase (GST), reducing the total number of ROS inside the cell through its thiol groups [34]. The genes encoding for these two enzymes contain ARE sequences in their promoter; therefore, their expression is directly activated by Nrf2. 

With this test, the absorbance value of a fluorescent compound obtained through the reaction of OPA with reduced glutathione was determined. 

**Figure 4:**
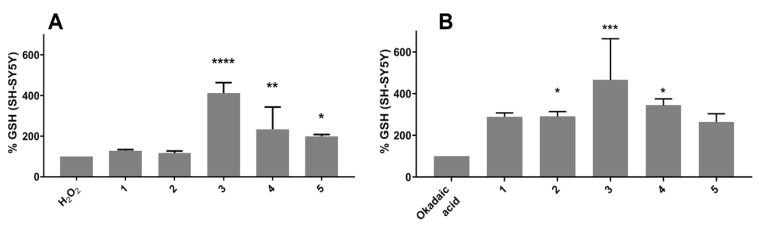
Effect of compounds **1**, **2**, **3**, **4**, and **5** (10 μM) on the GSH levels in SH-SY5Y cell line. Data are reported as percentages compared to cells treated only with H_2_O_2_ (100%) (**A**) and okadaic acid (100%) (**B**). The data are expressed as mean ± SD of two independent experiments. The statistical analysis was performed via two-way ANOVA, followed by Dunnett’s multiple comparisons test. * *p* < 0.05, ** *p* < 0.004, *** *p* < 0.001, **** *p* < 0.0001.

**Figure 5:**
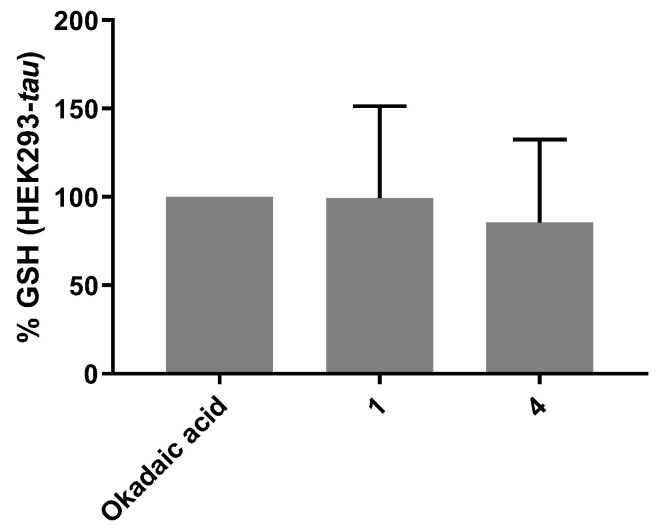
Effect of compounds **1** and **4** (10 μM) on the GSH levels in HEK293-*tau* cell line. Data are reported as percentages compared to cells treated only with okadaic acid (100%). The data are expressed as mean ± SD of independent experiments. The statistical analysis was performed using two-way ANOVA, followed by Dunnett’s multiple comparisons test. No significant results were obtained.

The assessment was carried out both on SH-SY5Y and HEK293-*tau*; in the first cell line, oxidative stress was induced alternatively with H_2_O_2_ 200 μM (Figure 4A) or okadaic acid 20 nM (Figure 4B), while HEK293-*tau* cells were treated only with 20 nM okadaic acid (Figure 5) because it is less aggressive than hydrogen peroxide on this cell line. Considering the results obtained in the cytoprotective test (see Section 3.3.2) carried out on SH-SY5Y cells, compounds **1**–**5** were tested on SH-SY5Y, whereas only compounds **1** and **4** were tested on HEK293-*tau* due to the higher cell viability showed in the cytotoxicity test after treatment with these compounds (see Section 3.3.1) compared to compounds **2**, **3**, and **5**. Through normalization with the values obtained with the bichinconinic acid (BCA) protein assay [35], the exact amount of glutathione per milligram of protein (GSH/mg protein) was calculated.

From the graph in Figure 4A, it is clear that compounds **3**, **4**, and **5** had an antioxidant effect on SH-SY5Y cells, with an increase in the GSH percentage, compared to control cells, of 312%, 124%, and 99%, respectively. When 25 nM okadaic acid was used as the toxic agent instead, all compounds gave excellent results with increased percentages of 190% (**1** and **2**), 366% (**3**), 244% (**4**), and 164% (**5**) (Figure 4B). Concerning the test carried out on the HEK293-*tau* cell line, neither compound **1** nor **4** showed significant antioxidant action compared to cells treated only with okadaic acid (Figure 5).

#### 3.3.4. LDH Liberation Test

**Figure 6:**
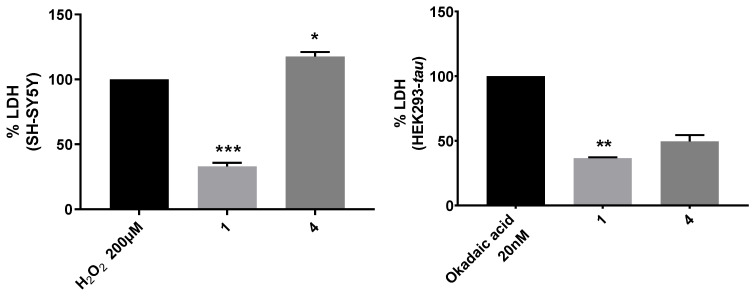
Analysis of the activity of the enzyme LDH released in the supernatant of SH-SY5Y (**left**) and HEK293-*tau* (**right**) cells after treatment with **1** and **4**. The results are reported as the percentage of LDH released compared to cells treated with only H_2_O_2_ or okadaic acid (100%). Data are expressed as averages ± SD of three independent experiments. The statistical analysis was performed via two-way ANOVA, followed by Dunnett’s multiple comparisons test. * *p* < 0.05, ** *p* < 0.01, and *** *p* < 0.001.

This test quantitatively evaluates lactate dehydrogenase (LDH), an enzyme present in the cellular cytoplasm that catalyzes the conversion of pyruvate into lactate, using nicotinammide adenine dinucleotide (NADH) as the cofactor [36]. If the cell suffers damage, such as that induced by oxidative stress, the plasma membrane is disrupted and the lactate dehydrogenase is released in an amount inversely proportional to NADH. Therefore, the lower the amount of LDH in the supernatant, the higher the protective and antioxidant action of the test compounds. This test measures the degree of conversion of NADH to NAD^+^ in an oxidizing environment. Since compounds **1** and **4** showed the best results in the cytoprotective test, to further elucidate their antioxidant pathway, they were assessed with additional tests. Like in the GSH assay, oxidative stress was generated using 200 μM H_2_O_2_ in SH-SY5Y and 20 nM okadaic acid in HEK293-*tau*. The results are reported as a percentage of LDH released from cells treated with the selected compounds, taking the mean absorbance values of cells treated only with H_2_O_2_ as 100% (Figure 6).

The data show that compound **1** reduced the release of LDH both in SH-SY5Y (67% compared to control) and in HEK293-*tau* (63.5% compared to control); compound **4**, on the other hand, did not protect against oxidative cell damage in the first cell line, while it lowered the release of LDH in HEK293-*tau* by 50.5%. These findings prompted us to evaluate the activation of the Nrf2 pathway. 

#### 3.3.5. Protein Analysis (Western Blotting)

The possible involvement of the transcriptional factor Nrf2 in the cytoprotective effect of **1** and **4** was evaluated. Moreover, considering the interconnection between the Nrf2 pathway and TAU [37], the total amount of phosphorylated Nrf2 in the nucleus of HEK239-*tau* was determined with Western blot, using β-actin as the control.

**Figure 7:**
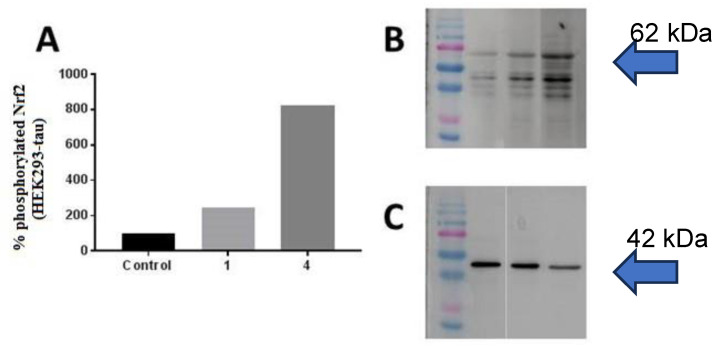
(**A**) Levels of phosphorylated Nrf2 in HEK293-*tau* cells treated with compounds **1** and **4**. Values are expressed as a percentage of untreated cells (control 100%). (**B**) Nrf2 62 kDa and (**C**) β-actin 42 kDa.

High levels of nuclear phosphorylated Nrf2 after treatment with **1** and **4** were detected (+145% and +721% respectively) (Figure 7), confirming that the protective properties shown by the two compounds against oxidant agents were not direct but exerted through the activation of the Nrf2-dependent enzymes.

## 4. Conclusions

Xanthones from *G. mangostana*, such as α-mangostin, γ-mangostin, garcinone D, and garcinone E, were reported to increase the protein levels of Nrf2 [38,39,40,41]. In this work, the cytoprotective action of five xanthones isolated from mangosteen against oxidative stress by modulating the production of GSH via Nrf2 transcription factor activation was assessed. Moreover, deeper insight into the mechanism through which xanthones allow Nrf2 protein accumulation and nuclear translocation was provided. The Nrf2 pathway regulates the expression of antioxidant genes, and Keap1 is a negative regulator of Nrf2, targeting it for degradation. So, the capability of compounds **1**–**5** to act as competitive non-covalent inhibitors for the binding site of Keap1 was investigated through molecular docking studies, which revealed that these compounds could interact with amino acids belonging to the five subpockets, competing with Nrf2 for the binding site, and so causing dissociation of Nrf2 from Keap1.

Prompted by the encouraging in silico results, the biological effects were determined through in vitro studies of the main effectors in the antioxidant response on human neuroblastoma from the SH-SY5Y line and human embryonic kidney cells from the HEK293-*tau* line. Compounds **1**–**5** showed a cytoprotective effect on SH-SY5Y in the presence of H_2_O_2_, with the best protective effect found 6 h after their administration.

Interestingly, 8-deoxygartanin (**3**) and garcinone D (**5**) showed direct antioxidant action by upregulating GSH turnover, and γ-mangostin (**1**) and demethylcalabaxanthone (**4**), despite being the most cytoprotective among the tested compounds, showed no significant improvement in the GSH levels. Moreover, demethylcalabaxanthone (**4**) could not lower the amount of lactate dehydrogenase (LDH), so its positive effect on cells treated with oxidative agents was probably due to an indirect effect involving the regulation of the expression of antioxidant enzymes. To demonstrate this, a quantification of the nuclear phosphorylated Nrf2 was performed, showing a marked increase in the translocated protein upon administration of **4**.

The present results extend and reinforce the notion that xanthones from *G. mangostana* possess protective effects against oxidative stress, which are related to their ability to increase Nrf2 transcription factor levels. In addition, the action of xanthones in disrupting the binding between Nrf2 and its natural inhibitor, Keap1, represents a promising strategy for the control of oxidative stress. All these findings highlight demethylcalabaxanthone (**4**) as a promising competitive inhibitor for Keap1, as it is capable of triggering the Nrf2-related antioxidant response.

## Figures and Tables

**Table 1:** List of the interactions of compounds **1**–**5** with Keap1. Interactions with residues that do not belong to the five subpockets are in bold. If multiple interactions were observed with the same residue, the total number is indicated in brackets.

Compounds	H-Bond	π-Cation	π-π Stacking	Hydrophobic
**1**	Ser363 and Asn414	Arg415 (2)	Tyr334 and Tyr525	Gly364, Arg380, Gly462, Arg483, Ser508, Gly509, Gln530, Ser555, Ala556, Tyr572, Phe577, Ser602, and Gly603
**2**	Ser363, Asn414, and Ser508	Arg415 (2)	/	Tyr334, Ser338, Gly364, Arg380, Asn382, Gly462, Gly509, Tyr525, Gln530, Ser555, Ala556, Tyr572, Phe577, and Ser602
**3**	Ser508	Arg415 (2)	Tyr525	Tyr334, Ser363, **Gly364**, Arg380, Asn382, Asn414, **Ile416**, Gly462, Arg483, Gly509, **Ala510**, Gln530, Ser555, Ala556, **Leu557**, Tyr572, Phe577, Ser602, and Gly603
**4**	Ser363 and Ser602	Arg415 (2)	Tyr334 and Tyr572	Gly364, Arg380, Asn382, Asn414, Gly462, Gly509, Ala556, Phe577, and Gly603
**5**	Ser363 and Ser508	/	Tyr525	Tyr334, **Gly364**, Arg380, Asn382, Asn414, Arg415, Ile461, Gly462, Phe478, Arg483, Gly509, Gln530, Ser555, Ala556, Tyr572, Phe577, Ser602, and Gly603

## Data Availability

The data presented in this study are available in the article.

## Supplementary Materials

The following supporting information can be downloaded at: https://www.mdpi.com/article/10.3390/antiox12111980/s1, Figures S1–S15: ESI/LTQOrbitrap and ^1^H and ^13^C NMR spectra of compounds **1**–**5**; Figure S16: results of the cytotoxicity test of compounds **1**, **2**, **3**, **4**, and **5** for SH-SY5Y and HEK293-*tau*.
