# Supplementary material for: Demethylcalabaxanthone from Garcinia mangostana Exerts Antioxidant Effects through the Activation of the Nrf2 Pathway as Assessed via Molecular Docking and Biological Evaluation
Source: Antioxidants (Basel). 2023 Nov 7;12(11):1980. doi: 10.3390/antiox12111980 (PMC10669650; doi:10.3390/antiox12111980)
Supplement: Supplementary file 1 [file antioxidants-12-01980-s001.zip › antioxidants-2683486-supplementary.pdf]

Demethylcalabaxanthone from *Garcinia mangostana* Exerts Antioxidant Effects through the Activation of the Nrf2 Pathway as Assessed via Molecular Docking and Biological Evaluation

Simona De Vita [1], Milena Masullo [1], Sabrina Grambone [1], Paloma Bermejo Bescós [2], Sonia Piacente [1,*] and Giuseppe Bifulco [1,*]

[1] Department of Pharmacy, University of Salerno, Via Giovanni Paolo II 134, 84084 Fisciano, Italy; sdevita@unisa.it (S.D.V.); mmasullo@unisa.it (M.M.) s.grambone@studenti.unisa.it (S.G.)
[2] Departamento de Farmacología, Farmacognosia y Botánica, Universidad Complutense de Madrid, 28040 Madrid, Spain; bescos@ucm.es
* Correspondence: piacente@unisa.it (S.P.); bifulco@unisa.it (G.B.); Tel.: +39-089-969763 (S.P.); +39-089-969741 (G.B.)

List of Figures

Figure S1. ESI/LTQOrbitrap spectrum of compound **1**
Figure S2. ^{1}H NMR spectrum of compound **1**
Figure S3. ^{13}C NMR spectrum of compound **1**
Figure S4. ESI/LTQOrbitrap spectrum of compound **2**
Figure S5. ^{1}H NMR spectrum of compound **2**
Figure S6. ^{13}C NMR spectrum of compound **2**
Figure S7. ESI/LTQOrbitrap spectrum of compound **3**
Figure S8. ^{1}H NMR spectrum of compound **3**
Figure S9. ^{13}C NMR spectrum of compound **3**
Figure S10. ESI/LTQOrbitrap spectrum of compound **4**
Figure S11. ^{1}H NMR spectrum of compound **4**
Figure S12. ^{13}C NMR spectrum of compound **4**
Figure S13. ESI/LTQOrbitrap spectrum of compound **5**
Figure S14. ^{1}H NMR spectrum of compound **5**
Figure S15. ^{13}C NMR spectrum of compound **5**
Figure S16**.** Results of the cytotoxicity test of compounds **1**-**5**

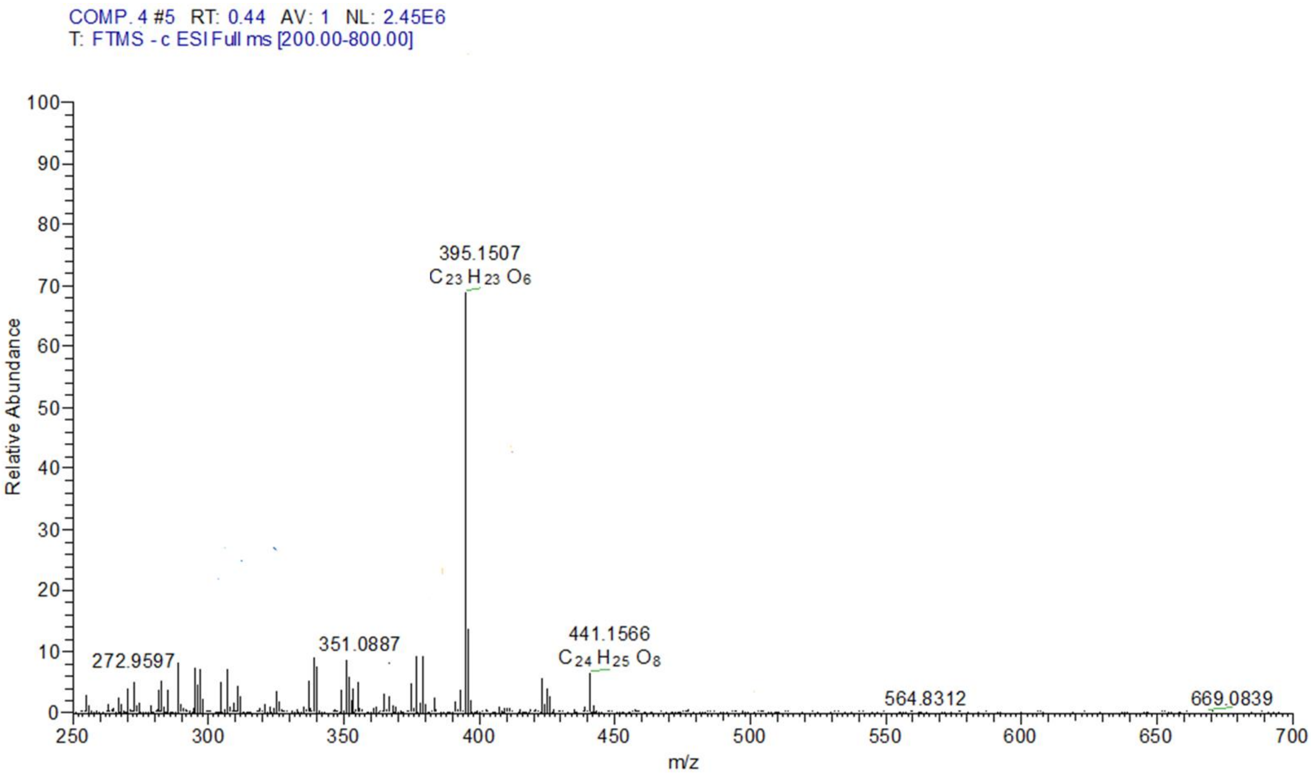

Figure S1. ESI/LTQOrbitrap spectrum of γ-mangostin (**1**), in negative ion mode

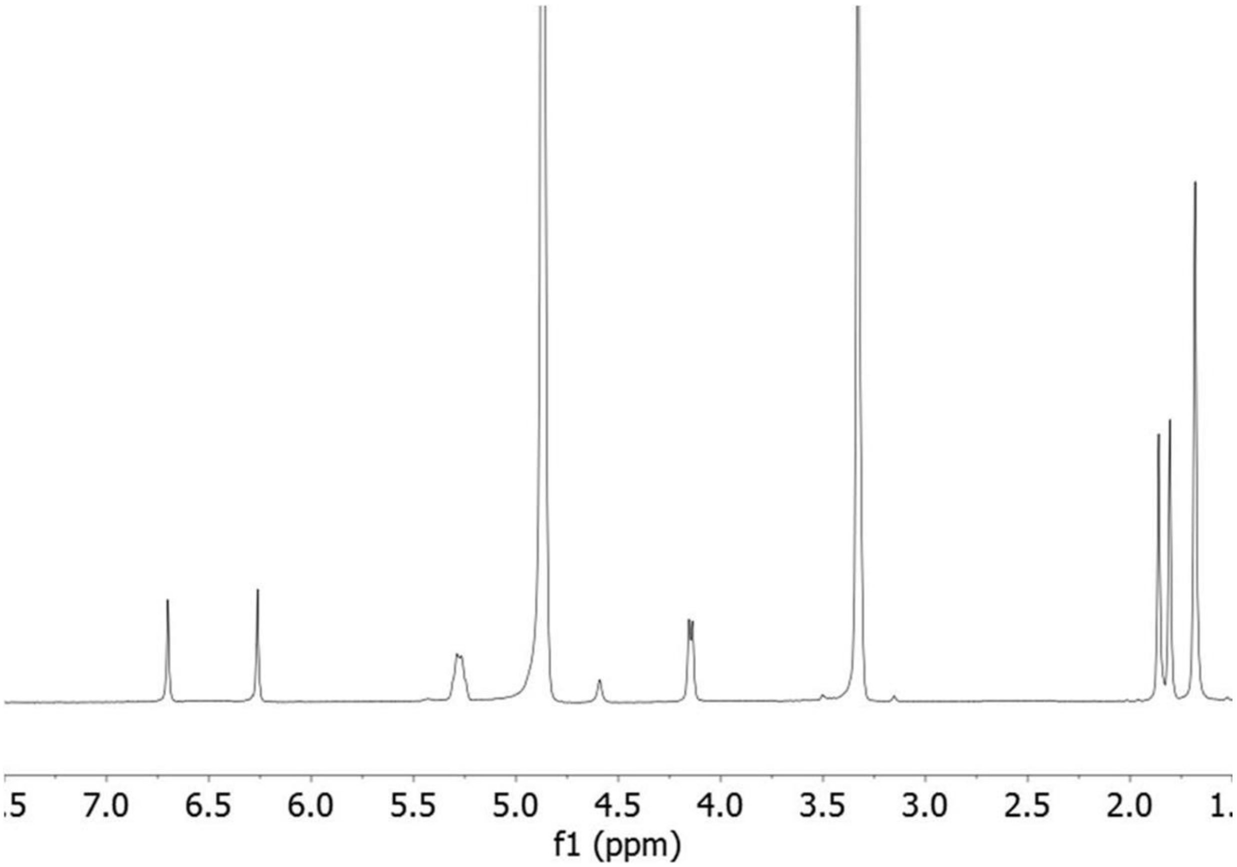

Figure S2. ^{1}H NMR spectrum (600 MHz, CD_3OD) of γ-mangostin (**1**)

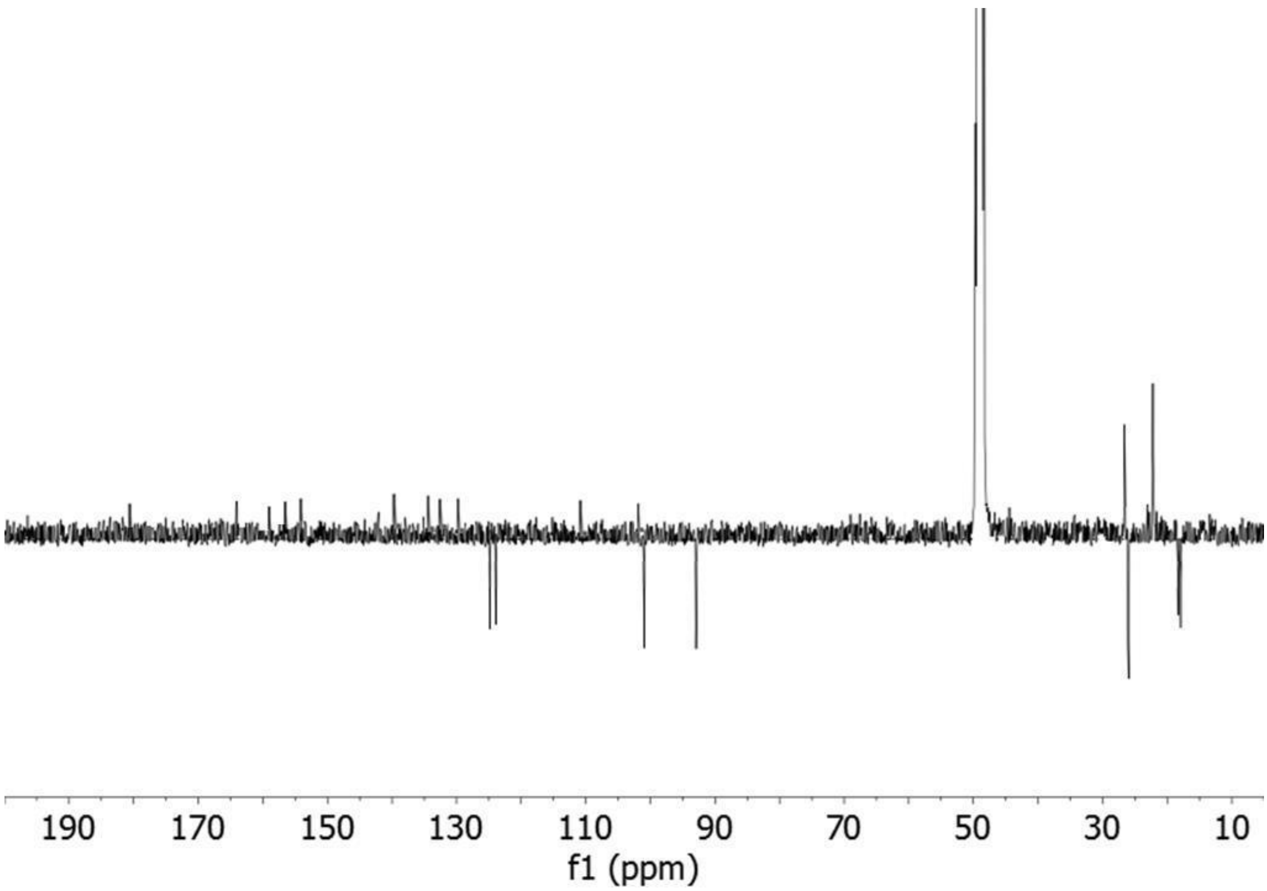

Figure S3. ^{13}C NMR spectrum (100 MHz, CD_3OD) of γ-mangostin (**1**)

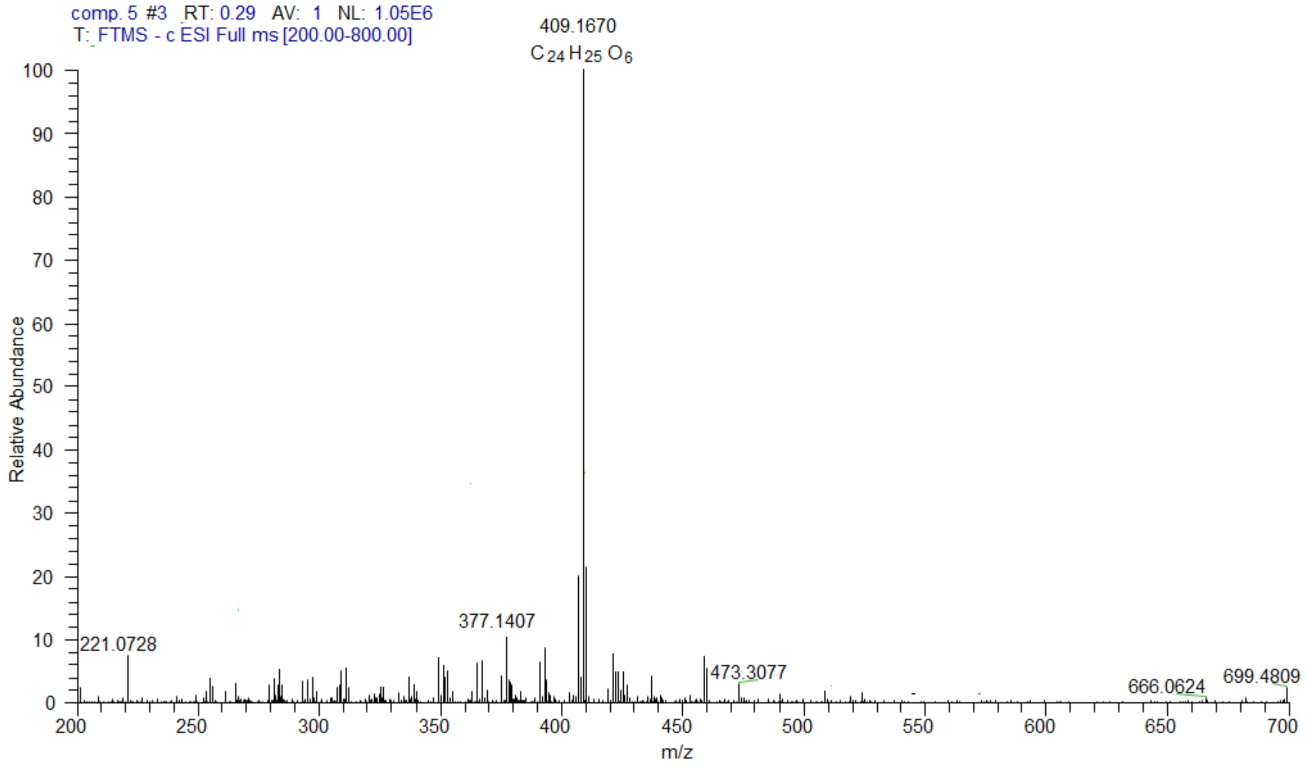

Figure S4. ESI/LTQOrbitrap spectrum of α-mangostin (**2**), in negative ion mode

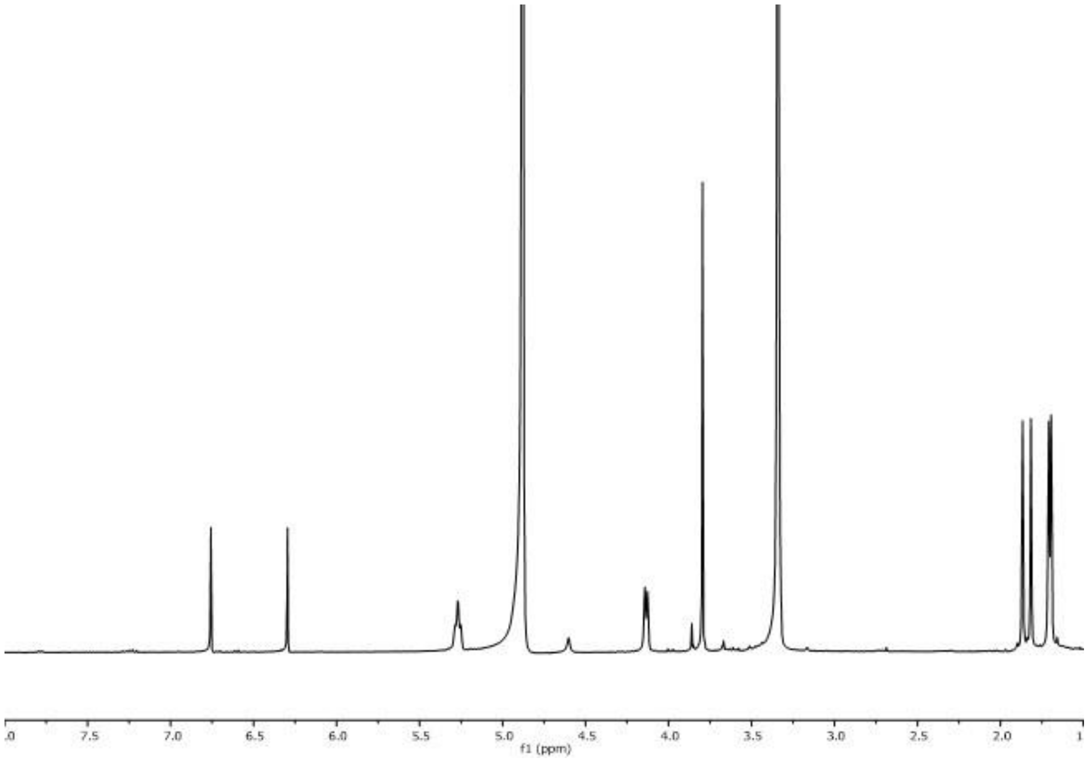

Figure S5. ^{1}H NMR spectrum (600 MHz, CD_3OD) of α-mangostin (**2**)

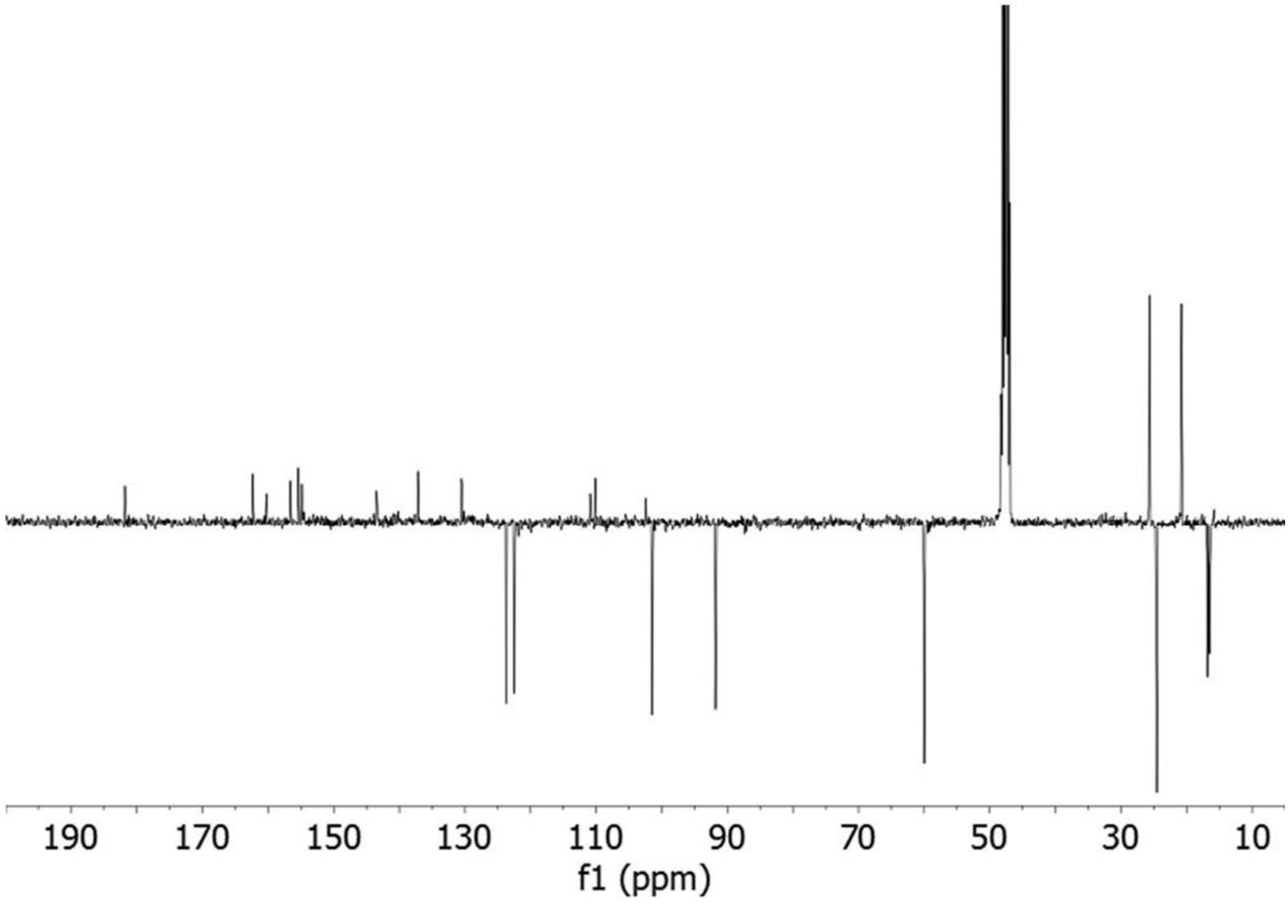

Figure S6. ^{13}C NMR spectrum (100 MHz, CD_3OD) of α-mangostin (**2**)

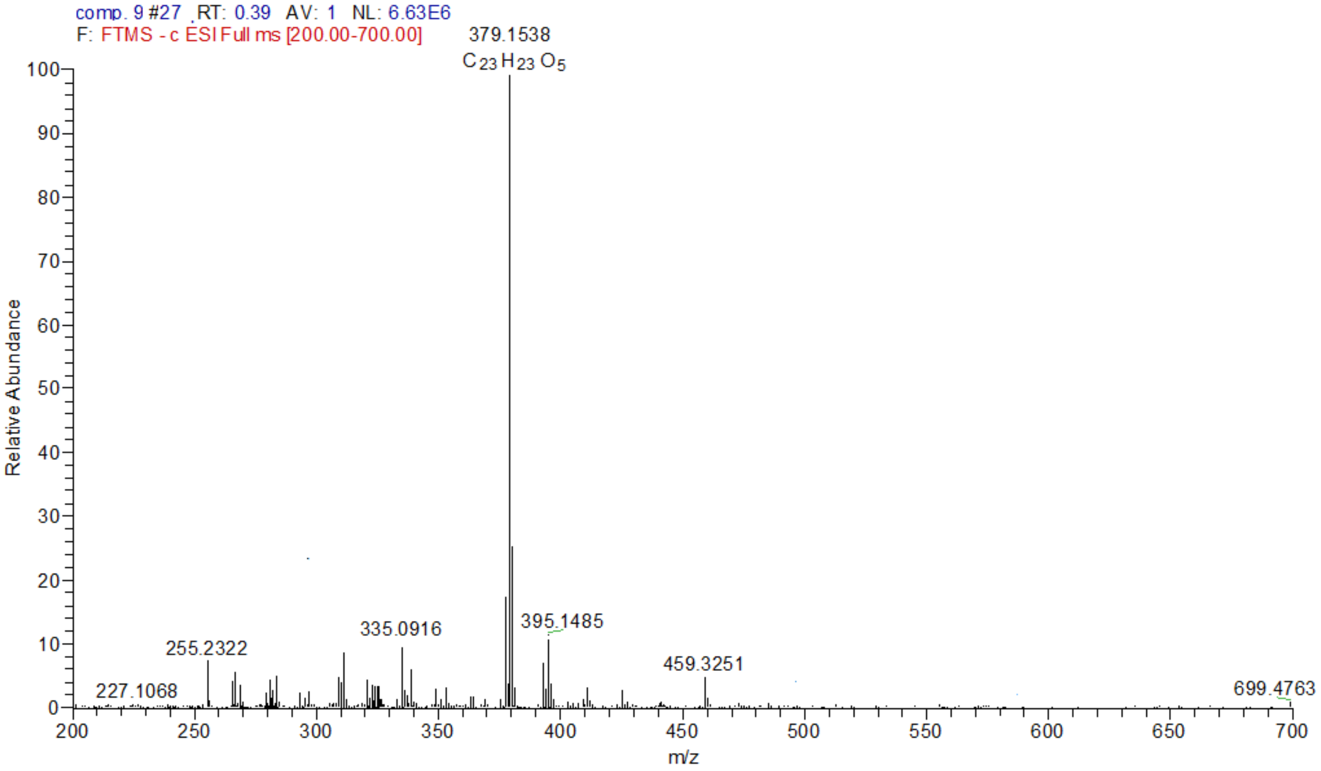

Figure S7. ESI/LTQOrbitrap spectrum of 8-deoxygartanin (**3**), in negative ion mode

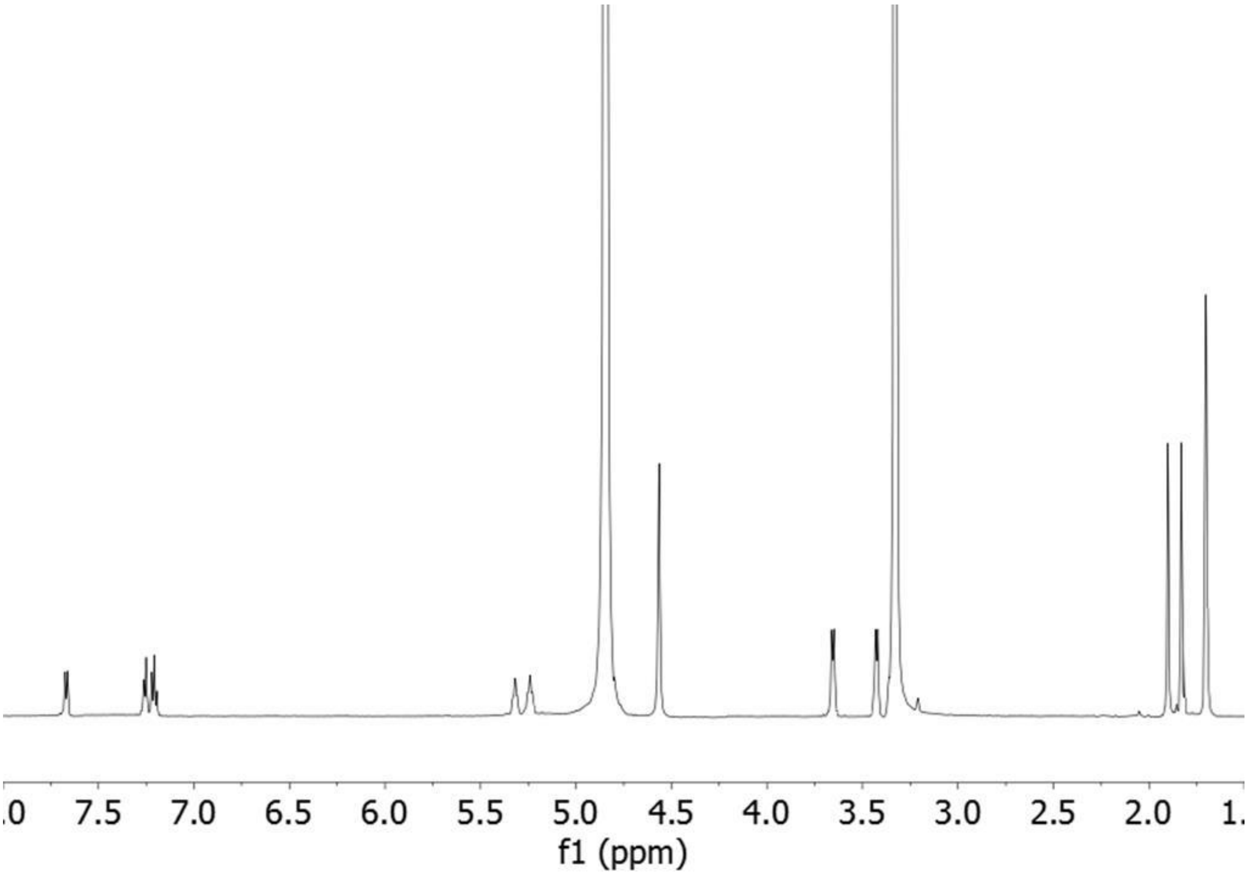

Figure S8. ^{1}H NMR spectrum (600 MHz, CD_3OD) of 8-deoxygartanin (**3**)

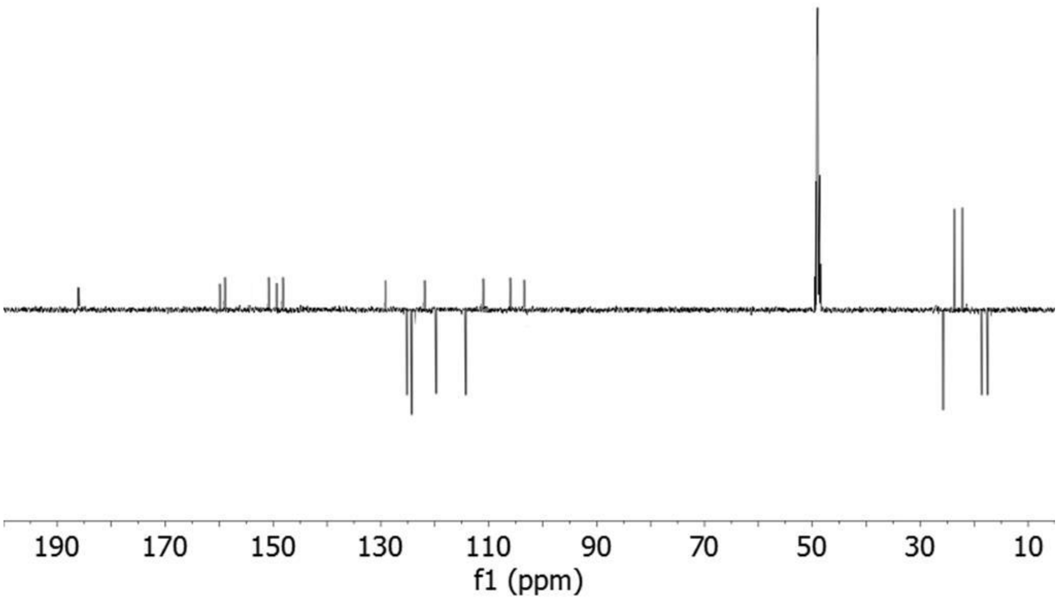

Figure S9. ^{13}C NMR spectrum (100 MHz, CD_3OD) of 8-deoxygartanin (**3**)

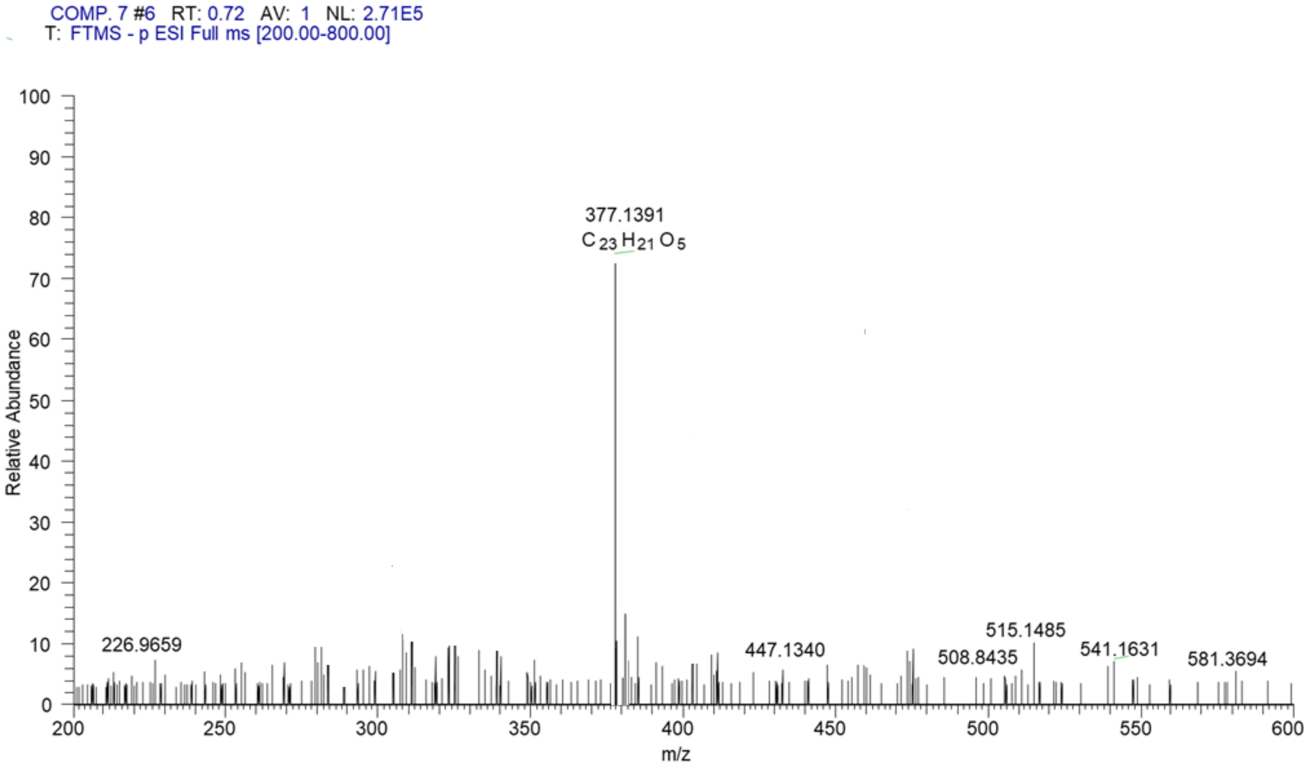

Figure S10. ESI/LTQOrbitrap spectrum of demethylcalabaxanthone (**4**), in negative ion mode

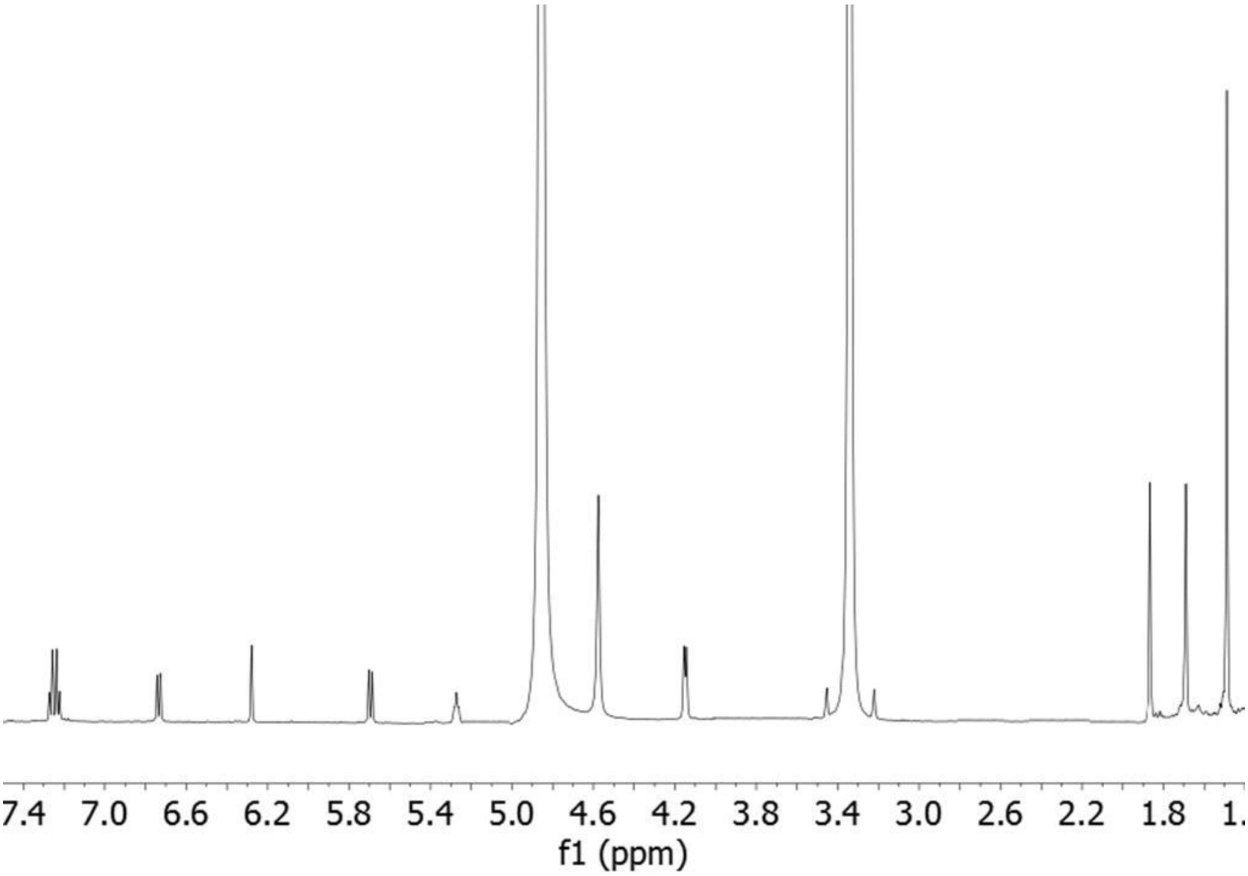

Figure S11. ^{1}H NMR spectrum (600 MHz, CD_3OD) of demethylcalabaxanthone (**4**)

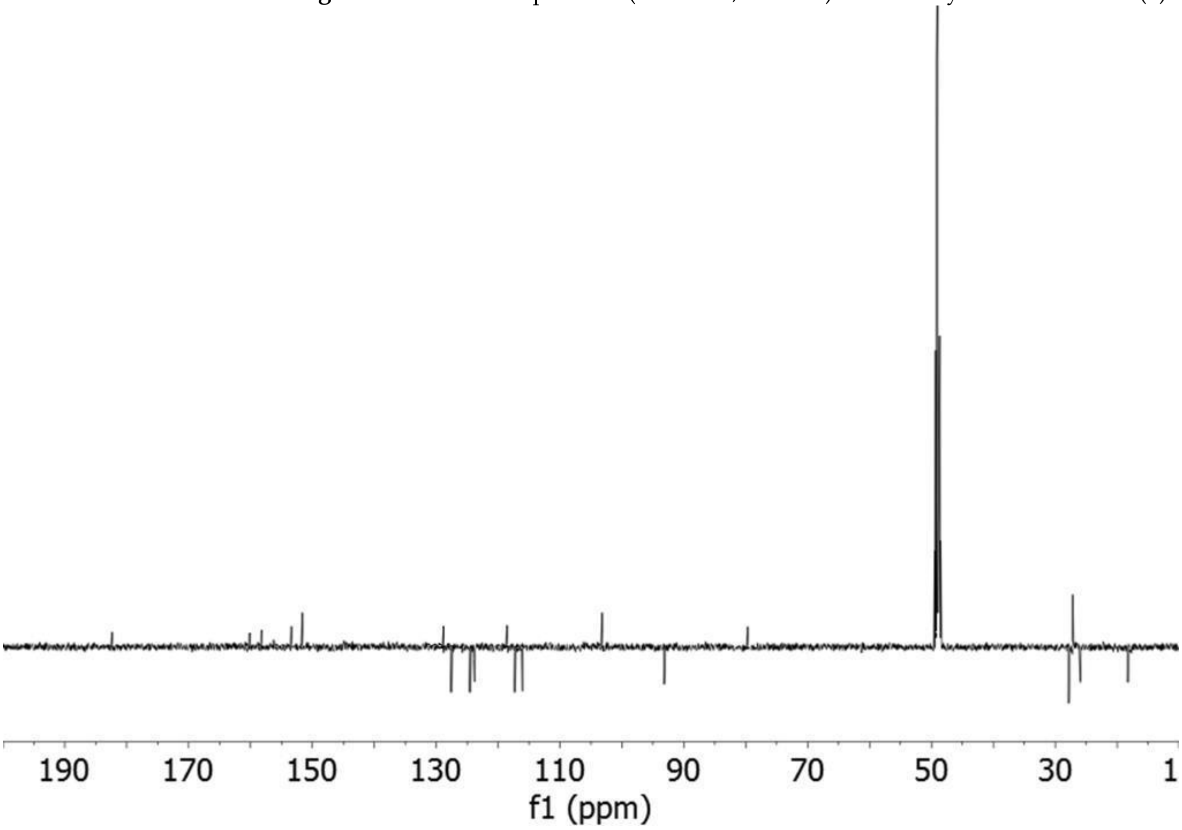

Figure S12. ^{13}C NMR (100 MHz, CD_3OD) spectrum of demethylcalabaxanthone (**4**)

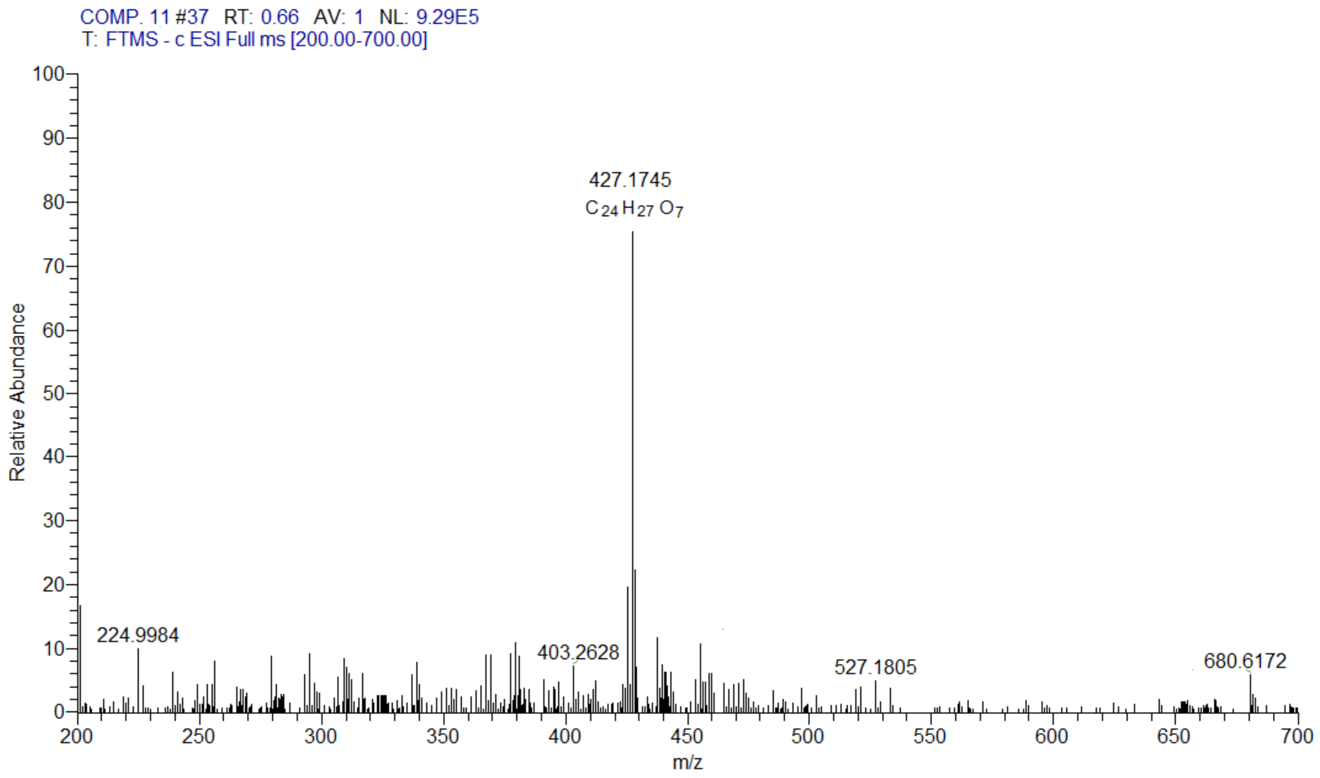

Figure S13. ESI/LTQOrbitrap spectrum of garcinone D (**5**), in negative ion mode

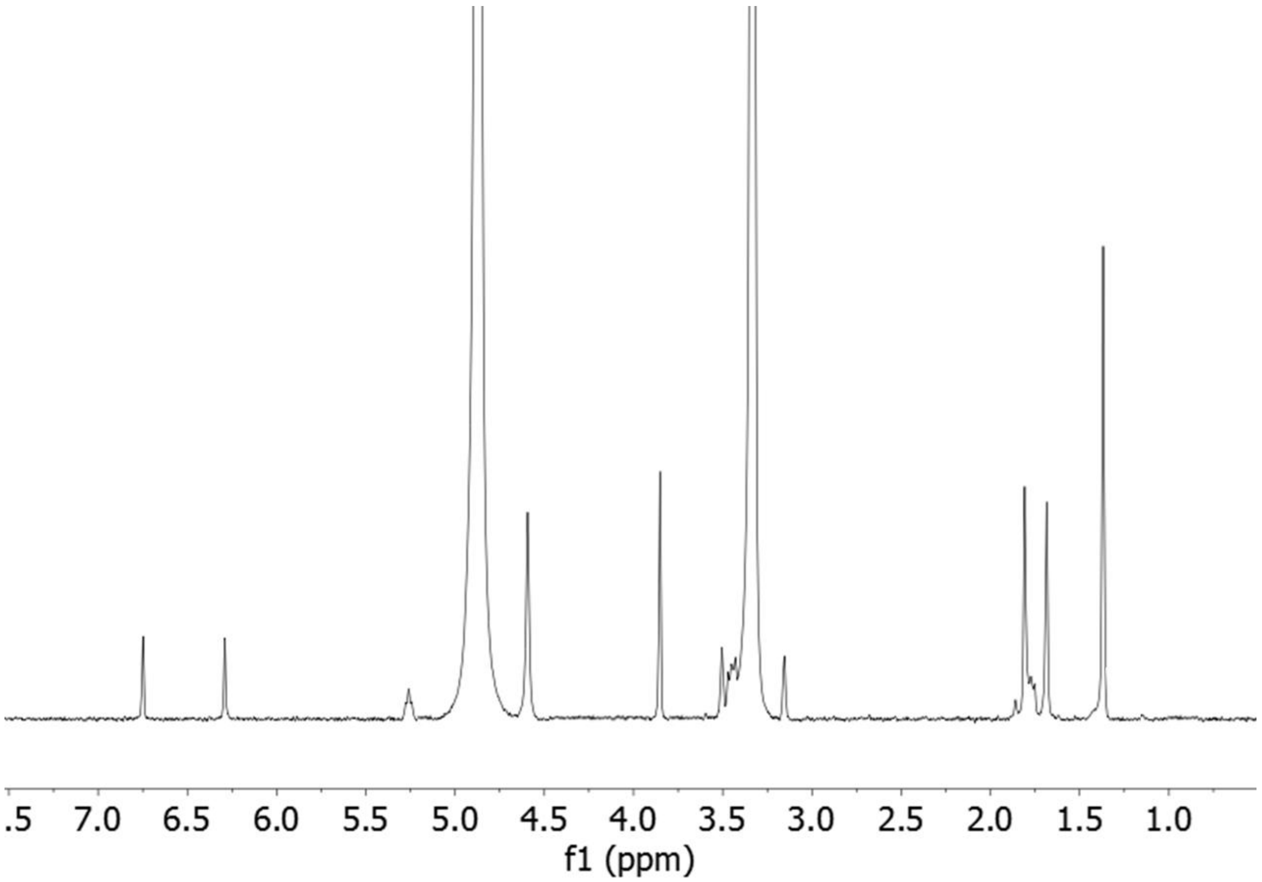

Figure S14. ^{1}H NMR spectrum (600 MHz, CD_3OD) of garcinone D (**5**)

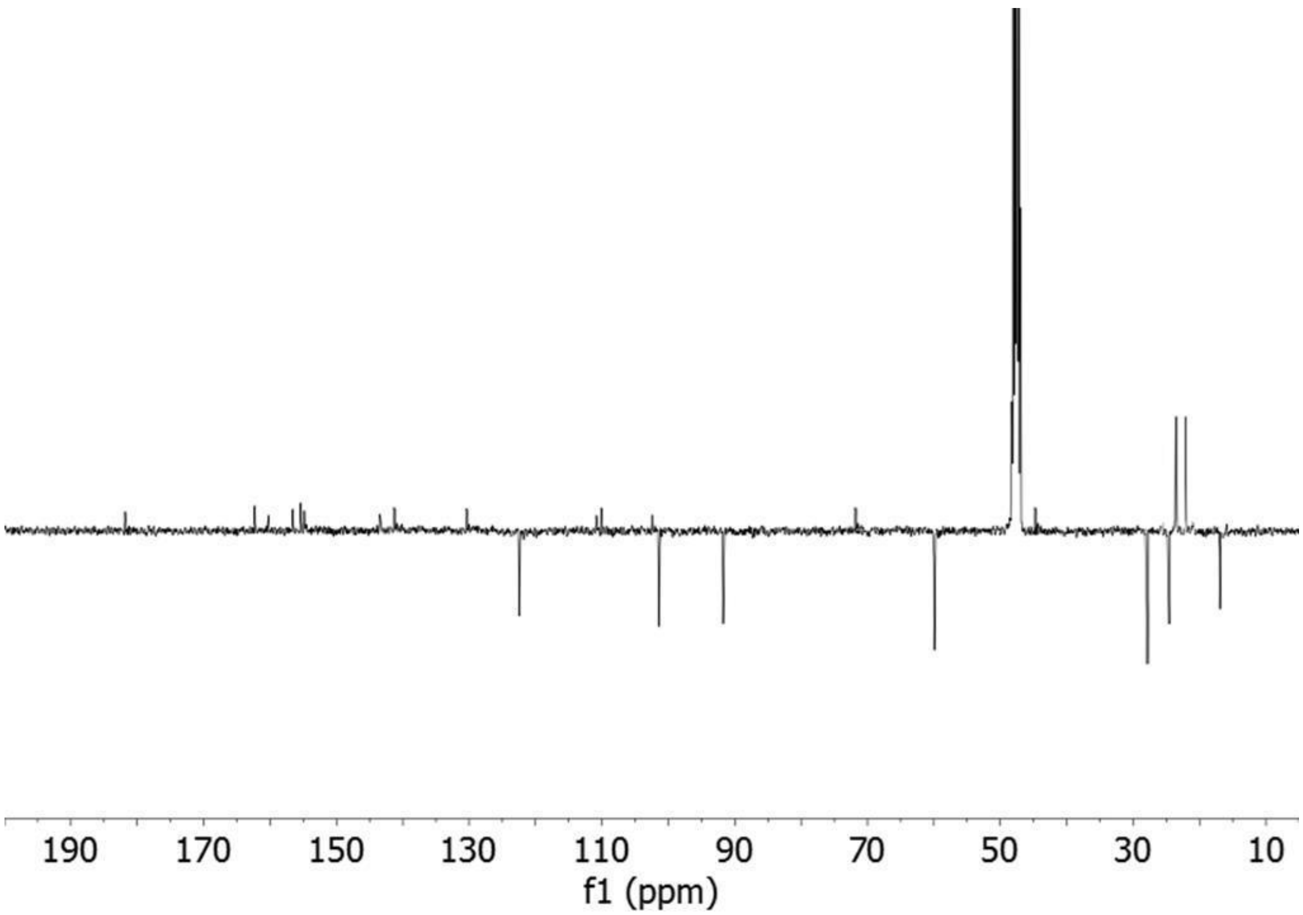

Figure S15. ^{13}C NMR (100 MHz, CD_3OD) spectrum of garcinone D (**5**)

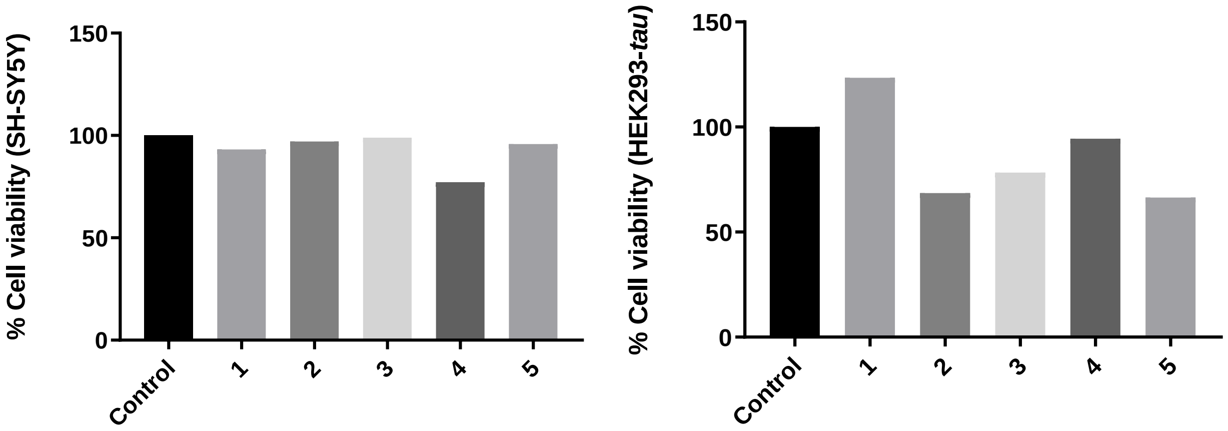

Figure S16. Results of the cytotoxicity test of compounds **1**, **2**, **3**, **4**, and **5** on SH-SY5Y and HEK293-*tau*, expressed as the percentage of viability compared to the control (100%).
